# Closing the Gaps in Menopause Care: Integrating Scalable System-Level Care Models for the Menopause Transition

**DOI:** 10.1016/j.jacadv.2025.102520

**Published:** 2026-03-18

**Authors:** Bede N. Nriagu, Faith E. Metlock, Lily Dastmalchi, Garima Sharma

**Affiliations:** Inova Schar Heart and Vascular, Inova Fairfax Medical Campus, Falls Church, Virginia, USA

**Keywords:** care models, integrated care, menopausal transition

The menopausal transition (MT) represents a pivotal stage in a woman’s life, with implications that extend beyond reproductive aging. This period is marked by profound hormonal, cardiovascular, metabolic, and psychosocial changes that shape long-term health outcomes.^1^ Although awareness of the MT has increased in recent years, gaps in knowledge and clinical practice persist among health care providers.^1^

Menopausal care is commonly delivered within primary care or obstetrics and gynecology settings,^2^ where limited time and training constrain comprehensive assessment. Some women turn to self-directed approaches, particularly those with the knowledge and resources to navigate available information,^3^ whereas those with complex symptoms or multiple comorbidities often require multidisciplinary care.^2^, ^3^, ^4^ Several care models have emerged to improve coordination and outcomes, yet the paucity of integrated, team-based care continues to result in conflicting recommendations, inconsistent counseling, and missed opportunities for prevention and long-term health optimization.

Despite growing recognition of menopause as a critical phase in women’s health, current evidence on care models remains largely descriptive, dominated by position papers and expert consensus rather than empirical evaluation.^1^, ^2^, ^3^ Comparative assessments across models are scarce, hindering identification of best practices and scalable strategies. Strengthening this evidence base is essential to guide workforce development, standardize care, and inform equitable health policy reform.

This viewpoint synthesizes current evidence on menopause care models, emphasizing multidisciplinary, empowerment centered, and technology-enabled approaches. By identifying shared principles and persistent challenges, it offers a unified perspective on integrating the most effective elements of current models to advance equitable and comprehensive menopause care.

## Care framework for women going through the menopause transition

The MT is marked by fluctuating and declining estradiol levels, leading to systemic symptoms and reduced ovarian function.^3^ Hypoestrogenism causes hot flushes, night sweats, sleep disturbance, mood changes, joint discomfort, and genitourinary symptoms such as vaginal dryness, dyspareunia, and urinary complaints.^3^ These symptoms can persist for years and significantly impact quality of life.

Beyond symptoms, the MT is associated with increased long-term health risks, including cardiovascular disease, adverse metabolic changes, bone loss, and musculoskeletal decline.^5^ Estrogen deficiency during the MT accelerates atherosclerosis, increases abdominal adiposity, and worsens lipid profiles, contributing to cardiovascular disease, the leading cause of death in women worldwide.^5^ As women spend more of their lives in postmenopausal, and with more than 1.2 billion women expected to be postmenopausal by 2030,^5^ menopause care is increasingly recognized as a clinical and public health priority. Integrated multidisciplinary models are emerging to address these needs, offering coordinated and scalable approaches to support long-term health.

### Multidisciplinary specialist care

Multidisciplinary models for menopause care are being adopted to improve coordination and address varied clinical needs during the transition. These models can be delivered in person, virtually, or through hybrid formats depending on patient preference and clinical need, expanding access and maintaining continuity across care environments.

#### In-person clinics

Dedicated in-person menopause clinics offer comprehensive face-to-face evaluation and treatment supported by structured referral pathways. In the United States, in-person multidisciplinary care models provide trusted, localized support with coordinated evaluation and management tailored to women’s needs.^6^ Another example, the interdisciplinary menopause clinics in Edmonton, Canada, show meaningful reductions in symptom burden and measurable improvements in well-being.^4^

#### Telehealth models

Telehealth-enabled menopause services provide flexible, affordable, and time-efficient access to care. Virtual consultations, whether individual or group-based, eliminate travel barriers and support continuity, with high levels of patient satisfaction reported.^7^ By extending reach across geographic and socioeconomic divides, telehealth promotes more equitable access to menopause expertise. Virtual-first services such as Midi Health and MyMenopauseRx in the United States deliver menopause specialist care across the country through telehealth platforms,^6^ making support accessible regardless of location.

#### Hybrid models

Hybrid care models integrate virtual access with timely in-person evaluation, allowing patients to receive remote care and attend clinic visits when needed. This approach improves coordination and timely access to specialist services. Carrot Fertility organizations in the United States exemplify this approach by combining virtual access with in-person clinical evaluation.^6^

Across all delivery formats, multidisciplinary collaboration remains central to helping women manage symptoms, improve quality of life, and maintain long-term health. Evidence from specialized interdisciplinary clinics demonstrates significant reductions in menopause-related symptom burden and improvements across vasomotor, psychosocial, physical, and sexual domains compared with waitlisted controls.^4^

### Integrative health care model

Described by Doubova et al,^8^ this model uses a multidisciplinary approach with coordinated input from a family doctor, nurse, and psychologist, together with shared decision-making. It incorporates a cognitive-behavioral framework delivered through individual and group counseling, emphasizing menopause education, lifestyle modification, and chronic disease prevention.^8^ By combining diverse expertise with active patient engagement, it promotes self-awareness, goal-setting, and problem-solving, supporting personalized continuous care.

### Tiered model of care

The British Menopause Society advocates a three-level framework aligned with patient complexity and clinician expertise.^9^ Level 1 involves all health care professionals, who should possess a basic understanding of menopause, its health impacts, and referral options.^9^ Level 2 includes primary-care clinicians with a special interest in menopause who provide consultations and coordinate care through defined referral pathways.^9^ Level 3 comprises specialists with advanced expertise in complex cases such as treatment failures, premature ovarian insufficiency, or hormone-dependent cancers.^9^ These specialists also lead local education initiatives, contribute to guideline development, and manage multidisciplinary management for cases beyond standard practice. Although formal evaluations are limited, this framework shows promise for coordinated context-sensitive care.

### Healthy menopause model

The European Menopause and Andropause Society Healthy Menopause Model integrates physical, psychological, and social dimensions to support quality of life in midlife women.^2^ It recognizes the growing burden of chronic conditions and promotes personalized, preventive care. At its core lies a collaborative triad comprising a lead clinician, specialist nurse, and the woman herself.^2^ Together, they develop individualized plans addressing short-, medium-, and long-term goals that reflect each woman’s health status, values, and lived experience. This model underscores the importance of comprehensive, integrated expertise to optimize health across the MT.

### Empowerment model

This model reframes menopause as a stage for self-management and shared decision-making rather than a hormonal deficiency.^3^ Grounded in the World Health Organization definition of health empowerment, it positions women as active partners in their care, supported by clinicians who provide empathetic listening, balanced information, and evidence-based guidance.^3^ It emphasizes educational, stigma reduction, and workplace inclusivity to enhance knowledge, confidence, and autonomy.^3^ By prioritizing empowerment over medicalization, this approach promotes holistic well-being and equity in menopause care. Qualitative studies of cognitive behavioral therapy for vasomotor symptoms show that women who adopt self-management strategies report greater knowledge, acceptance, and control.^4^ By centering autonomy and inclusivity, it complements clinical and digital innovations, shifting from reactive symptom treatment to proactive woman-centered health promotion.

### Human rights-based approach

The human rights–based model frames menopause management within the principles of equity, dignity, and justice in health.^10^ It asserts that all women, including those in prisons or other marginalized settings, deserve care equivalent to those available in the community.^10^ This model emphasizes age- and gender-sensitive, nondiscriminatory services that safeguard physical and psychological well-being, including access to diagnosis, treatment, essential medicines, and supportive environments. Grounded in international human rights law, this approach defines menopause care as both a clinical obligation and a matter of social justice, ensuring that even the most vulnerable women receive comprehensive, dignified, and equitable care.

### Integrated Framework for Women’s Midlife Health

Menopause care requires flexible strategies tailored to diverse symptoms, health profiles, and social contexts. Models have developed worldwide, shaped by local health care systems, resources, and patient needs.^2^^,^^3^^,^^7^, ^8^, ^9^ Despite advances across diverse care models, persistent challenges remain, including workforce shortages,^2^ gaps in medical training in menopause management within postgraduate curricula,^1^ and the absence of standardized evaluation metrics.^3^ Addressing these system-level gaps requires identifying best practices across models, developing quality indicators, and advancing policies that incentivize team-based, equitable care. Promising strategies such as hybrid integration, community partnerships, and tiered referral frameworks offer scalable pathways to bridge specialty silos and embed menopause care as a cornerstone of women’s health.

**Figure 1:**
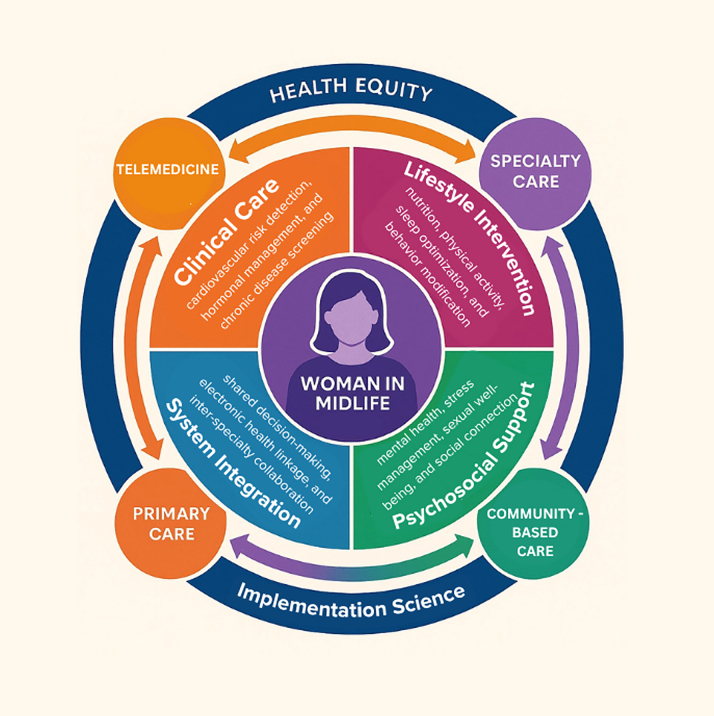
Integrated Framework for Menopause Care: Centering the Midlife Woman Through Equity, Empowerment, and System Integration

This synthesis culminates in a comprehensive care framework (Figure 1) that centers the woman in midlife and connects multidisciplinary, lifestyle, psychosocial, and system-level supports through the guiding principles of health equity and implementation science. As reflected in this framework, comprehensive menopause care integrates 4 interdependent domains: 1) clinical care that includes hormonal management, cardiovascular risk detection, and chronic disease prevention; 2) lifestyle interventions emphasizing nutrition, physical activity, sleep, and behavior modification; 3) psychosocial support addressing mental health, stress, and social connection; and 4) system integration that ensures coordination across primary, specialty, and community-based care.

Anchored in implementation science, the framework emphasizes adaptability, evaluation, and sustainability across diverse care settings. Health equity, represented as the encompassing outer layer, underscores that each component must reach all women, particularly those historically underserved. Together, these interconnected domains provide a scalable, preventive model for menopause care that advances equity and supports lifelong cardiovascular, metabolic, and psychosocial well-being.

## Funding support and author disclosures

The authors have reported that they have no relationships relevant to the contents of this paper to disclose.
